# Research Progress on the Combination of Quorum-Sensing Inhibitors and Antibiotics against Bacterial Resistance

**DOI:** 10.3390/molecules29071674

**Published:** 2024-04-08

**Authors:** Jiahao Wang, Xingyue Lu, Chenjie Wang, Yujie Yue, Bin Wei, Huawei Zhang, Hong Wang, Jianwei Chen

**Affiliations:** Key Laboratory for Green Pharmaceutical Technologies and Related Equipment of Ministry of Education & Key Laboratory Pharmaceutical Engineering of Zhejiang Province & College of Pharmaceutical Science & Collaborative Innovation Center of Yangtze River Delta Region Green Pharmaceuticals, Zhejiang University of Technology, Hangzhou 310014, China; jiahao2021302@163.com (J.W.); luxingyue123456@163.com (X.L.); 2111230070063@zjut.edu.cn (C.W.); 1112107014@zjut.edu.cn (Y.Y.); binwei@zjut.edu.cn (B.W.); hwzhang@zjut.edu.cn (H.Z.)

**Keywords:** antibiotics, combination, effect, quorum-sensing inhibitors, mechanism

## Abstract

Bacterial virulence factors and biofilm development can be controlled by the quorum-sensing (QS) system, which is also intimately linked to antibiotic resistance in bacteria. In previous studies, many researchers found that quorum-sensing inhibitors (QSIs) can affect the development of bacterial biofilms and prevent the synthesis of many virulence factors. However, QSIs alone have a limited ability to suppress bacteria. Fortunately, when QSIs are combined with antibiotics, they have a better therapeutic effect, and it has even been demonstrated that the two together have a synergistic antibacterial effect, which not only ensures bactericidal efficiency but also avoids the resistance caused by excessive use of antibiotics. In addition, some progress has been made through in vivo studies on the combination of QSIs and antibiotics. This article mainly expounds on the specific effect of QSIs combined with antibiotics on bacteria and the combined antibacterial mechanism of some QSIs and antibiotics. These studies will provide new strategies and means for the clinical treatment of bacterial infections in the future.

## 1. Introduction

**Figure 1:**
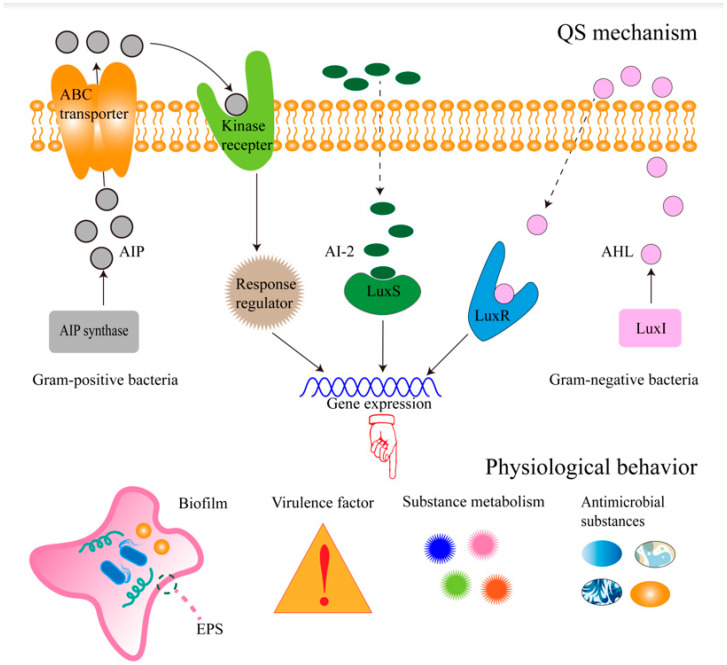
Regulatory mechanism and influence of QS. When the bacterial density reaches the threshold, the extracellular QS signal molecules AHL, AIP, and AI-2 bind to the corresponding receptor proteins, promote the expression of QS-related genes, and ultimately cause biofilm formation and virulence factor expression, antimicrobial substance production, and substance metabolism.

Microorganisms secrete signal molecules to the extracellular environment during growth, such as QS molecules. The signal molecules accumulate with the increase in population density, and when the response threshold is reached, the signal molecules bind to the receptor and regulate gene expression and physiological activities through a series of cascade reactions, such as biofilm formation, virulence factor expression, and antibacterial substance synthesis. This phenomenon is called QS. Many published studies have examined the QS system of bacteria. The activation of the QS system of Gram-negative bacteria is mainly based on *N*-acyl homoserine lactone (AHL) as a QS signal molecule, and the AHL-mediated QS system has a variety of signal receptor homologs, including LuxI/R, LasI/R, RhlI/R, and AfeI/R [1]. Taking the LuxI/R system as an example, the LuxI enzyme promotes the synthesis of AHLs; when the bacterial density reaches the threshold, AHLs bind to their receptor protein LuxR to form a complex. The complex is then combined with the target gene promoter sequence to promote the expression of the target gene and the physiological activity of the bacteria [2]. The activation of the QS system in Gram-positive bacteria mainly depends on cyclic oligopeptide small molecule compounds (autoinducer peptide, AIP). AIP synthase promotes the synthesis of AIPs, and when the bacterial density reaches the threshold, the extracellular AIPs can be combined with histidine kinase to promote the phosphorylation of histidine kinase. Then, the phosphorylated histidine kinase can promote the signal cascade reaction and finally induce the attachment of bacterial biofilm or the production of toxins and extracellular proteases [3]. In addition, QS can also exist between Gram-positive and Gram-negative bacteria, which is mainly promoted by the interspecies signal molecule Autoinducer-2 (AI-2) encoded by the *luxS* gene. When the bacterial density reaches the threshold, AI-2 binds to the LuxS receptor protein and promotes the relationship and activity between bacteria [4]. Figure 1 shows the regulation mechanism and influence of QS.

**Figure 2:**
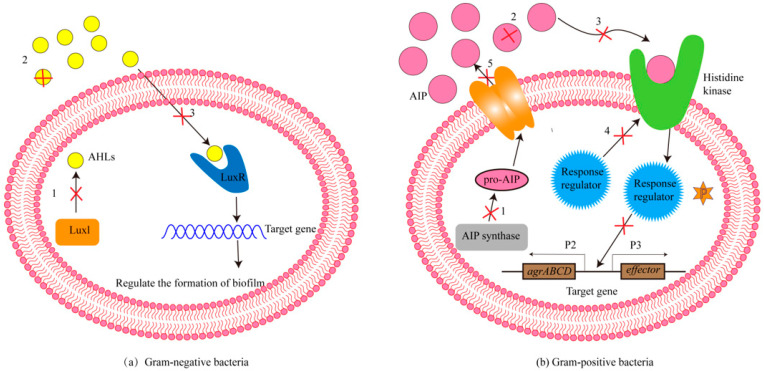
The mechanisms behind QSIs controlling the biofilm formation of Gram-negative bacteria (**a**) and Gram-positive bacteria (**b**). The mechanisms behind QSIs controlling bacterial biofilm formation mainly include the following: (1) hindering the biosynthesis of AIs; (2) inactivating AIs via AHL-lactonase, oxidoreductase, antibodies, or other substances; (3) using AI antagonists to interfere with signal receptors; (4) using the interference response regulator and creating the interference signal cascade reaction; and (5) inhibiting AIs efflux, reducing extracellular AIs accumulation, and thereby inhibiting intercellular signal transduction.

Bacterial infection is the leading cause of human death, and people often use antibiotics to kill pathogens. Unfortunately, due to the overuse and abuse of antibiotics, the resistance of bacteria to existing antibiotics is increasing [5,6]; therefore, it is urgent to find a new method to deal with drug-resistant bacteria. The regulation of the QS system, an important intercellular communication system, is performed by the release of autoinducers (AIs). AIs can track changes in bacterial density, control the expression of bacterial-related genes [7,8], and adjust the bacteria to adapt to the environment by controlling bioluminescence, pigment synthesis, biofilm formation, and the secretion of virulence factors [9]. Interestingly, the formation of bacterial biofilms causes drug resistance in bacteria [10]. The bacterial biofilm is composed of a cell community that accumulates or adheres to the cell surface, and the cell community is embedded in an extracellular polymeric substance (EPS) matrix. This EPS contains extracellular DNA (eDNA), proteins, lipids, polysaccharides, biopolymers, and divalent cations, and provides a barrier against antibiotics [11,12]. Therefore, how to eliminate the adverse effects of biofilm on antibiotics is an important topic, and to address this issue, some researchers have examined drug combinations. The fractional inhibitory concentration index (FICI) is usually used to measure whether there is interaction between drug combinations. FICI < 0.5 suggests that the drug combination has a synergistic effect. Meanwhile, 0.5 ≤ FICI ≤ 1 suggests that the drug combination has an additive effect (additive to synergistic). In contrast, 1 < FICI < 4 suggests that the drug combination is irrelevant (additive to antagonistic), and FICI ≥ 4 suggests that the drug combination has an antagonistic effect [13,14]. At present, studies have shown that QSIs can be used as antibacterial synergists to prevent the growth of bacterial biofilms by inhibiting bacterial QS systems; the specific mechanism behind this is shown in Figure 2. For Gram-negative bacteria, there are three main ways to inhibit the formation of bacterial biofilm. These include the following: (1) the biosynthesis pathway of the LuxI enzyme to signal molecule AHLs can be hindered; (2) the AHL lactone enzyme, oxidoreductase, antibody, and other types of QSIs can directly lead to the inactivation of signal molecule AHLs; and (3) signal molecule antagonists can compete with AHLs for LuxR receptor protein to inhibit the expression of QS genes. For Gram-positive bacteria, there are five main ways to inhibit the formation of bacterial biofilms, including: (1) blocking the synthesis pathway of AIP synthase to AIP; (2) promoting the inactivation of AIP; (3) hindering the binding of AIP to extracellular histidine kinases, resulting in the inability of histidine residues of histidine kinases to phosphorylate and thereby inhibiting downstream signaling pathways; (4) interfering with response regulators, and thus interfering with signal cascades; and (5) blocking AIP from entering the cell through transporters. As a result, extracellular AIP cannot accumulate to the threshold, and thus cannot initiate the QS system [15,16,17]. Therefore, some research has examined the idea of combining QSIs and antibiotics as a new sterilization strategy. In this paper, the combined application of QSIs with a variety of common antibiotics and the possible synergistic antibacterial mechanism are discussed.

## 2. Combination of Aminoglycoside Antibiotics and QSIs

Aminoglycoside antibiotics can treat patients with persistent Gram-negative bacterial infections, tuberculosis, and cystic fibrosis [18]. However, with the emergence of more drug-resistant bacteria, the application of aminoglycoside antibiotics is also limited. Therefore, the combination of drugs, especially the combination of antibiotics and QSIs, is likely to become a new means to fight bacterial infection [19,20].

### 2.1. Combination of Tobramycin and QSIs

In order to explore whether naturally active small molecule coumarins and their derivatives can improve the antibacterial activity of tobramycin (TOB), TOB alone (*P. aeruginosa*, MIC, 2 μg mL^−1^) or combined with 200 μg mL^−1^ of farnesifrol A, B, C (**1**–**3**), gummosin (**4**), and 4-farnesyloxycoumarin (**5**) were used to treat *Pseudomonas aeruginosa* (*P. aeruginosa*), respectively. The findings indicated that the proportion of surviving bacteria treated with TOB alone was 86%. After binding to coumarin derivatives (**1**–**5**), the proportion of viable bacteria was 27%, 27%, 34.6%, 66.6%, and 69%, respectively. In addition, molecular docking results also showed that coumarin derivatives could be used as PqsR inhibitors to inhibit the biofilm and virulence factors of *P. aeruginosa*. This further shows that coumarin derivatives are likely to indirectly improve the antibacterial efficiency of TOB by inhibiting the QS system. However, whether coumarin derivatives assist TOB antibacterial in other ways is worth further study [21].

In previous studies, we found that coumarin natural compounds have QSI activity, and hydroxamic acid derivatives can chelate iron. Based on this research progress, coumarin and hydroxamic acid derivatives were connected via chemical synthesis. Compound **6** (*P. aeruginosa* biofilm, IC_50_ = 3.6 μM) with dual biofilm inhibitory activity was obtained, and its synergistic antibacterial ability with TOB and ciprofloxacin (CIP) was explored. It was found that **6** could increase the antibacterial activity of TOB by 200-fold. A further mechanism study found that **6** could not only inhibit biofilm formation by inhibiting the QS system of *las* and *pqs* or competing with siderophore pyoverdine (Pvd) for siderophore receptor FpvA, but also inhibit the expression of bacterial motility genes and multidrug resistance (MDR) efflux system-related genes (*mexA*/*mexE*). Whether the biofilm is inhibited or the expression of efflux pump genes (*mexA*/*mexE*) is down-regulated, the antibacterial effect of antibiotics will be greatly improved. The above results not only prove the antibacterial synergistic potential of **6**, but also reveal the mechanism of **6**, which provides a more reference for the future study of QSIs combined with antibiotics [22].

As mentioned above, curcumin and its derivatives exhibit good QSI activity. However, studies have found that many QSIs have limited application due to poor water solubility and low bioavailability. Therefore, some researchers have used nanomaterials to load and improve the availability of drugs [23]. In particular, the polyamidoamine dendrimer (PAMAM) loaded with curcumin (Cur) and biotin-polyethylene glycol-polylysine (biotin-PEG-PLys) modified with 2,3-dimethylmaleic anhydride (DA) can be combined into Cur-DA NPs via electrostatic interaction, and Cur-DA NPs was further modified with anti-CD54 to obtain anti-CD54@Cur-DA NPs. Subsequently, the synergistic anti-biofilm activity of Cur-DA NPs and antibiotics was further explored. The results showed that TOB treatment alone (20 μg mL^−1^) reduced the biofilm biomass of *P. aeruginosa* by 59.3%. However, biofilm biomass treated with Cur-DA NPs combined with TOB decreased by up to 87.3%. Furthermore, reverse transcription-polymerase chain reaction (RT-PCR) research revealed that the expression of inner membrane efflux pump-related genes (*mexD* and *mexE*), outer membrane protein–related genes, and membrane fusion protein-related genes were down-regulated, which may explain the synergistic anti-biofilm activity of Cur-DA NPs and antibiotics. More importantly, a mouse model of chronic pulmonary infection was used to evaluate whether Cur could enhance antibiotic therapy in vivo. The outcomes demonstrated that the sterilization rate of TOB treatment alone was 37.7%, while Cur-DA NPs combined with TOB treatment increased the sterilization rate to 87.3%. Interestingly, the sequential treatment of anti-CD54@CurDA NPs combined with TOB resulted in a further reduction in bacterial colonies in the lungs up to 98.6%. The above results demonstrate the potential of drug delivery through nanomaterials, which provides a new method to fight bacterial infection in the future [24].

Based on the core skeleton of 3-hydroxypyridin-4(1*H*)-one, a series of novel 3-hydroxypyridin-4(1*H*)-one derivatives with 4-aminomethyl-1,2,3-triazole linkers were designed and synthesized. The triazole linker was the key component for the derivatives to maintain the inhibitory activity of pyocyanin, and **7** had good *pqs* inhibitory activity (IC_50_ = 3.7 μM) and pyocyanin inhibitory activity (IC_50_ = 2.7 μM). Subsequently, the synergistic antibacterial effect of **7** with antibiotics was further explored, and it was found that compared with TOB treatment alone, the combination of compound **7** (10 μM) with 1/2, 1/4, or 1/8 of the minimum inhibitory concentration (MIC) of TOB created a notable decline in the bacterial viability of *P. aeruginosa* PAO1, FB, 1121, and 1129 strains. The in vivo effect of **7** was further explored through the *Caenorhabditis elegans* (*C.elegans*) infection model, and the survival rate of infected nematodes treated with a sub-inhibitory concentration of TOB alone was 18.1%. However, **7** (10 and 50 μM) combined with TOB enhanced the survival rate of *C.elegans* to 46.7% and 82.3%, respectively. The above in vitro and in vivo studies have shown that **7** has the potential as an antibacterial synergist, but whether it can be used in clinical practice may require more research [25].

Non-steroidal anti-inflammatory drugs of ketoprofen derivatives **8**, **9**, and **10** can act on the *pqs* system of *P. aeruginosa*. The use of TOB alone did not affect the cells surrounded by the *P. aeruginosa* biofilm matrix, but the antibacterial effect of TOB was significantly improved when it was combined with **8**, **9**, and **10**. Among them, the antibacterial effect of the combination of TOB can be increased to more than 50%. In fact, combined with previous and current research studies, ketoprofen derivatives can destroy bacterial biofilm matrix (especially EPS and protein). This may indicate that **8**, **9**, and **10** are likely to promote TOB to play a better role by destroying the biofilm barrier of bacteria. However, there are few studies on the activity of ketoprofen derivatives binding antibiotics; in the future, the interaction between the two can be further studied using an animal infection model and mechanism [26].

The design of drugs based on receptors is also a common method used for obtaining new drugs [27]. Compound **11** is a quinolone signal receptor inverse agonist obtained via chemical synthesis. Studies have shown that compound **11** (IC_50_ = 0.346 μM) can inhibit the release of eDNA from the *P. aeruginosa* PA14 biofilm matrix. Compared with 0.5 μg mL^−1^ TOB treatment alone, TOB combined with **11** (1 μM or 10 μM) had a better antibacterial effect on PA14. The combined treatment resulted in a more than 3-fold decrease in colony-forming units (CFU). In addition, TOB and **11** showed synergistic activity in the primary and secondary infection sites in the mouse infection model, which further indicated that compound **11** had the potential to become an antibacterial synergist [28].

**Figure 3:**
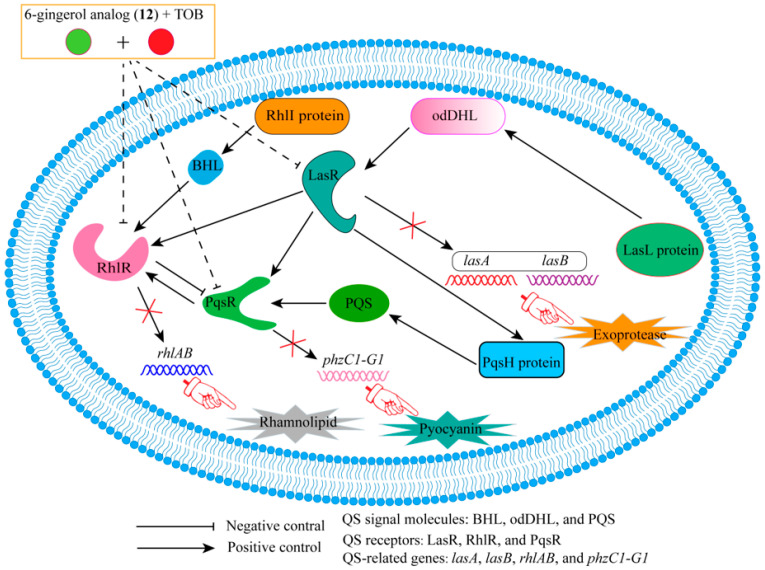
The mechanism of the combination of **12** and TOB in changing QS gene expression and virulence. The combination of compound **12** with TOB showed antagonistic binding activity to LasR, RhlR, and PqsR receptors. TOB enhanced the ability of compound **12** to compete with QS signal molecules to bind to QS receptors. When **12** binds to QS receptors, it causes the expression of QS- related genes to be unable to generate exoprotease, rhamnolipid, and pyocyanin.

6-gingerol is an active product derived from ginger. Based on the structure of 6-gingerol, the 6-gingerol analog (**12**) can be obtained by further modification. During in vitro studies, the biofilm inhibition rate of *P. aeruginosa* treated with 1 μM **12** combined with 0.63 μM TOB was as high as 60%, and FICI was 0.39. In addition, with **12** or TOB treatment alone, the carbohydrate content of the biofilm decreased by 15% to 24%, and the protein content decreased by 12% to 26%. However, the combination of the two resulted in a further decrease in the carbohydrate and protein content of the *P. aeruginosa* biofilm (50% to 52%). More importantly, QS receptor binding experiments and real-time reverse transcriptase quantitative polymerase chain reaction (RT-qPCR) analysis showed that the binding of compound **12** to TOB showed antagonistic binding activity to LasR, RhlR, and PqsR receptors. TOB was likely to increase the binding ability of compound **12** to QS receptors LasR, RhlR and PqsR, which in turn hindered the binding of BHL (*N*-butyryl-homoserine lactone) produced by the RhlI enzyme to the RhlR receptor, the binding of odDHL (*N*-3-oxo-dodecanoyl-_L_-homoserine lactone) produced by the LasL enzyme to the LasR receptor, and the binding of PQS (2-heptyl-3-hydroxy-4-quinolone) produced by the PqsH protein to the PqsR receptor. As a result, the expression of QS-related genes (*lasA*, *LasB*, *rhlAB*, and *phzC1-G1*) was inhibited, which ultimately affected the formation of exoprotease, rhamnolipid, and pyocyanin (Figure 3 shows the mechanism of the combination of **12** and TOB to change QS gene expression and virulence). On the other hand, in vivo studies have found that the survival rate of *P. aeruginosa*-infected *Tenebrio molitor* larvae (*T. molitor* larvae) treated with **12** or Tob is 40–60%. In comparison, combining the two can raise the survival rate of the *T. molitor* larvae to 90%. In particular, the combination of the two in human lung epithelial cells not only promotes cell proliferation, but also does not interfere with lactate dehydrogenase (LDH) release, which further illustrates the possibility of combining the two in the clinic [29].

In order to explore the combination effect of QSIs combined with antibiotic therapy and the importance of early treatment, mice infected with *P. aeruginosa* were treated with furanone C-30 (**13**), ajoene (**14**), or horseradish juice extract combined with TOB, respectively. The results showed that after using **13** or TOB alone, the CFU of *P. aeruginosa* in each implant was almost higher than 10^5^, but the combination of the two reduced the CFU of *P. aeruginosa* in each implant to around 10^5^. Similarly, combining **14** or horseradish juice extract with TOB reduced the CFU of *P. aeruginosa* in each implant. In addition, there were significant differences in the effects of early treatment (from the day before infection to 1 day after infection) or late treatment (from the 11th day to the 13th day after infection) between the control group and the combined administration group. In the early treatment, the effect in the combined administration group was ~100- to 150-fold that of the therapeutic effect in the control group, but with the late treatment, the combined administration group showed a weaker therapeutic effect, and the effect was only 1.3- to 4-fold that of the control group. In addition, previous studies have also shown that the extract of **14** or horseradish juice is likely to improve the antibacterial effect of TOB by inhibiting the biofilm. This study proves the feasibility of the combined effect as well as proving the importance of early treatment, which provides new ideas for future research [30,31,32]. 

*N*-(2-pyrimidinyl) butyramide (**15**) has a structure similar to that of the quorum-sensing signal molecule. In order to further explore whether **15** and antibiotics had synergistic anti-biofilm activity, *P. aeruginosa* biofilms were treated with **15**, antibiotics, and **15** + antibiotics under aerobic and anaerobic conditions, respectively, and observed using confocal laser scanning microscopy (CLSM). The results showed that **15** and antibiotics did have synergistic anti-biofilm activity, especially under aerobic conditions. Although biofilm formation was inhibited after TOB treatment alone (4–6 log CFU/cm^2^), the biofilm inhibition rate was higher after combined treatment with **15** (<1 log CFU/cm^2^). Similar results can be seen under anaerobic conditions. In addition, the destroyed biofilm was observed by CLSM, and it can be speculated that **15** may promote the antibacterial effect of antibiotics such as TOB by destroying the biofilm structure. Therefore, combining **15** and antibiotics may become a new means to fight bacterial infection. However, in vivo research is lacking, so it is necessary to explore the best conditions for combined application [33].

Compound **16** is a low-molecular-weight quaternized chitosan derivative (QAL), which also has anti-biofilm activity. Studies have shown that the combination of 0.037 mg mL^−1^ of **16** and 0.25 μg mL^−1^ of TOB had a significant synergistic antibiofilm effect on four strains of *P. aeruginosa* (PA ATCC, PA W4, PA C2118, and PA B910). The combination therapy could reduce the biofilm formation of *P. aeruginosa* by about 90% when compared with the control; moreover, a sub-inhibitory concentration of **16** may enhance the antibacterial action of TOB by disrupting the bacterial membrane, encouraging TOB diffusion through the outer membrane, or increasing the active absorption of bacterial biofilm [34].

Oleic aldehyde coumarate (OALC) (**17**) is a naturally active molecule derived from *Dalbergia trichocarpa* which can induce a reduction of in EPS production and the destruction of the biofilm structure. In particular, TOB treatments alone can only kill 36 ± 4% of *P. aeruginosa* biofilm cells, and the combination of the two can kill 90 ± 5% of biofilm cells. Once the biofilm is destroyed, antibiotics are likely to pass through the biofilm matrix more easily, thereby exerting a better antibacterial effect [35].

Further modification of the parent nucleus structure of itaconimide and citronimide can lead to compounds **18** and **19** with stronger QSI activity. This study found that the treatment with **18** alone can only kill the biofilm base population, but cannot kill the biofilm surface, and the results of TOB treatment alone are just the opposite. Fortunately, the combination of the two can eradicate the entire biofilm population, which indicates that **18** and TOB have synergistic anti-biofilm activity. However, **19** did not show synergistic anti-biofilm activity with TOB due to its poor water solubility. Luckily, some studies have found that drug loading via nanomaterials will likely improve the water solubility and utilization of drugs. Therefore, further research can be carried out from this point of view [36,37].

Cinnamic acid and its derivatives are naturally active molecules derived from Chinese herbal medicine. It was found that 4-dimethylaminocinnamic acid (**20**) and 4-methoxycinnamic acid (**21**) can lead to changes in the structure and permeability of biofilms, making it easier for TOB to penetrate biofilms and exert antibacterial effects. In particular, 0.3 μg mL^−1^ of TOB (*Chromobacterium violaceum* (*C. violaceum*), MIC, 0.5 μg mL^−1^) combined with **20** (200 μg mL^−1^) or **21** (100 μg mL^−1^) treatment can cause the biofilm of *C. violaceum* ATCC12472 to decrease by 63% and 79%, respectively. In addition, metabolomics analysis revealed that **20** and **21** resulted in a decrease in ethanolamine (biofilm component) and D-proline (osmotic pressure regulator), which also explained why **20** and **21** are promising TOB adjuvants [38].

The Food-and-Drug-Administration-approved clinical drug albendazole (**22**) has been shown to have QSI activity. It can act acts on both CviR and LasB receptor proteins of *C. violaceum* and *P. aeruginosa*. CLSM showed that the biofilm of *P. aeruginosa* plasB-gfp (ASV) treated with 75 μM of **22** and TOB (80 mg mL^−1^) was more severely damaged than that treated with **22** or TOB alone; this not only illustrates the potential of **22** as an antibiotic adjuvant, but also shows that it is indeed a good idea to explore by screening the new activity of known drugs [39].

**Figure 4:**
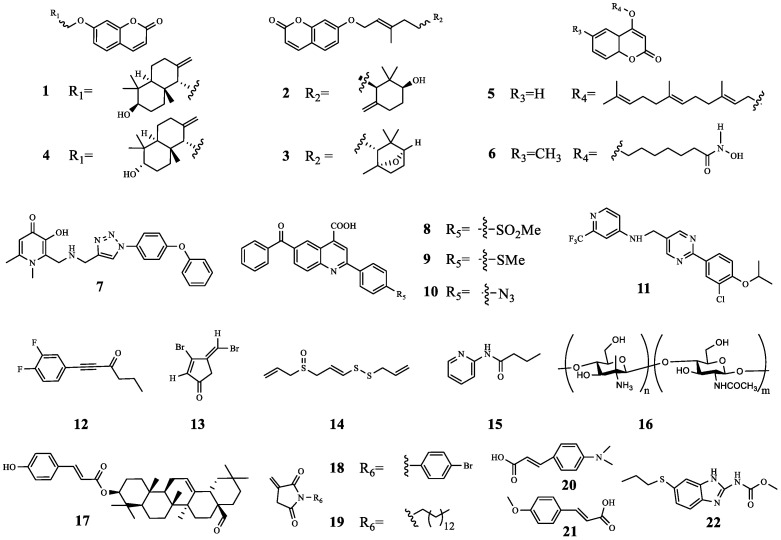
The chemical structures of compounds **1**–**22**.

In summary, combination with QSIs has a better therapeutic effect than TOB alone. In particular, the disclosure of the synergistic effect of compounds **6** and **12** with TOB has allowed more researchers to gain a deeper understanding of QSIs. However, the shortcomings of the above studies are that some studies only involve in vitro studies and do not involve in vivo studies; therefore, this also provides a new goal and direction for future research and a new method for treating antibacterial infection. The chemical structures of compounds **1**–**22** (Figure 4) and the results of their combined antibacterial effects with TOB are summarized in Table 1.

### 2.2. Combination of Gentamicin and QSIs

Plumbagin (**23**) is mainly derived from the roots of Plumbaginaceae plants. It can act on the *las* system and inhibit bacterial biofilm production. The therapeutic effects of the sub-minimum inhibitory concentration (sub-MIC) dose of **23** (<50 μg mL^−1^) combined with a sub-MIC dose of gentamicin (GM) (1/2 MIC to 1/10 MIC) on *P. aeruginosa* (MTCC 424 and MTCC 2488) have been studied. The first three groups acted on *P. aeruginosa* MTCC 424, and group A was treated with 30 μg mL^−1^ of **23** + 1.6 μg mL^−1^ of (1/6 MIC) GM. Group B was treated with 35 μg mL^−1^ of **23** + 2.5 μg mL^−1^ of GM (1/4 MIC), and group C was treated with 40 μg mL^−1^ of **23** + 1.25 μg mL^−1^ of GM (1/8 MIC). These findings demonstrate that *P. aeruginosa* biofilm inhibition rates in groups A, B, and C were 65%, 58%, and 60%, respectively. The latter three groups acted on *P. aeruginosa* MTCC 2488, and could also produce different degrees of inhibition. In addition, this study also proved that the combination of the two could eliminate the biofilm activity and inhibit the expression of virulence factors. When **23** was combined with GM, the FICI against *P. aeruginosa* MTCC 424 was 0.192, and the FICI against *P. aeruginosa* MTCC 2488 was 0.485. This further indicates that the two have synergistic antibacterial activity. Therefore, combination therapy is likely to be a new strategy used to reduce the formation of bacterial biofilms and provide new ideas for combating the occurrence of drug resistance [40].

Etrasimod (APD334) (**24**) is a sphingosine-1-phosphate receptor (S1PR) modulator of *Staphylococcus aureus* (*S. aureus*). In order to explore whether **24** (*S. aureus* ATCC 25923, MIC, 2.3–4.6 μg mL^−1^) combined with a variety of antibiotics has a synergistic anti-*S. aureus* effect, some 96-well plates were used to configure the mixture of the two, and the antibiotic concentration started from 2-fold MIC and then was diluted 3–4 times in turn. The results showed that **24** and GM had a synergistic effect on *S. aureus*, and the FICI was 0.5. Compared with the minimum inhibitory concentration (MIC) of GM treatments alone, the MIC of GM in the combination of **24** and GM was reduced by 4-fold. The above data indicate that combination therapy is likely to reduce the occurrence of drug resistance and reduce the side effects caused by high doses of GM by reducing the dose of GM [41].

Tannic acid (**25**) is a QSI derived from plants. The combination antibacterial action of antibiotics and **25** can be investigated using the antibiotic disc-diffusion method. The findings demonstrated that the combination of GM and **25** was more effective in inhibiting *Salmonella enterica* Paratyphi 3336 (*S.* Paratyphi 3336) than antibiotics alone (an inhibition zone from 18.7 mm to 27.0 mm). Similar results were also found for *Salmonella enterica* Typhi 950 (*S.* Typhi 950) (an inhibition zone from 20.3 mm to 25.0 mm); in addition, studies have shown that **25** can reduce the resistance of *S.* Typhi 950 and *S.* Paratyphi 3336 cells to GM and restore the use of GM once more for infections caused by drug-resistant *S.* Typhi 950 and *S.* Paratyphi 3336. In addition, EPS quantitative analysis showed that **25** could significantly reduce the EPS content (35–50%) of *S.* Typhi 950 and *S.* Paratyphi 3336, resulting in the destruction of the biofilm structure, which is likely to cause more bacteria to be unable to resist GM, thereby restoring the antibacterial effect of GM [42].

**Figure 5:**
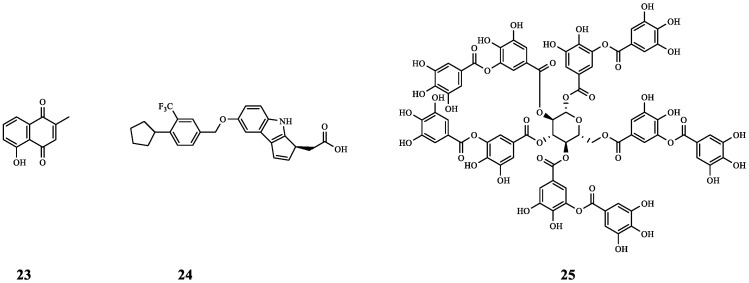
The chemical structures of compounds **23**–**25**.

The above examples show that **23**–**25** has the potential to become an antibacterial synergist of GM and clarify the synergistic effect of **23** and **24** combined with GM against *P. aeruginosa* and *S. aureus*, respectively, providing a new means for the future fight against *P. aeruginosa* or *S. aureus* infection. The chemical structures of compounds **23**–**25** (Figure 5) and the results of their combined antibacterial effects with GM are summarized in Table 2.

## 3. Combination of β-Lactam Antibiotics and QSIs

Previous studies have found that β-lactam antibiotic resistance is closely related to biofilm barrier, β-lactamase production, and efflux pump activity. In order to resist bacterial resistance, a series of anti-biofilm agents, β-lactamase inhibitors, and efflux pump inhibitors have been produced [43,44]. Previous studies have found that QSIs can inhibit biofilm production by inhibiting the QS system, and some QSIs can also inhibit β-lactamase activity or efflux pump gene expression [45,46]. Therefore, QSIs and β-lactam antibiotics are likely to become a new means to fight bacterial infection.

### 3.1. Combination of Ceftazidime and QSIs

The synergistic antibacterial effect of TOB combined with curcumin (**26**) analogues has been described above. Here, we further explore whether **26** can improve the antibacterial effect of Ceftazidime (CAZ). Researchers have explored the synergistic inhibitory effect of **26** combined with CAZ and CIP on QS-related genes and virulence characteristics of *P. aeruginosa* at sub-inhibitory doses. It was found that combined with CAZ or CIP, it could significantly reduce swarming and twitching motilities. The addition of **26** reduced the MIC of CAZ and CIP. Moreover, **26** showed a synergistic effect (FICI = 0.26) with CAZ and an additive effect (FICI = 1.0) with CIP; this once again shows the potential of **26** and its derivatives to be used as antibacterial synergists [47]. At present, the research on **26** is extensive. Whether based on the structural modification of **26** or the combination with nanoparticles, it provides a new idea for combating bacterial infection.

Trp-containing peptides are effective antibacterial agents. Studies have found that almost all tryptophan-containing peptides have synergistic or additive effects with antibiotics against multidrug-resistant *P. aeruginosa* (MRPA0108). In particular, L1W has a synergistic effect with CAZ or piperacillin (PRL), and the FICI values are 0.43 and 0.49, respectively. Similarly, L12W also synergizes with CAZ or PRL, and the FICI values are 0.23 and 0.28. More importantly, L1W and L12W not only decreased the activity of β-lactamase by 4-fold and 1.8-fold, respectively, but also significantly inhibited the relative expression of efflux pump genes (*OprM*, *MexX*, and *MexA*). Both the decreased β-lactamase activity, and the inhibition of efflux further improved the antibacterial effect of antibiotics. The above results proved the potential of Trp-containing peptides as antibiotic adjuvants, and subsequent studies may be more convincing if they can increase the activity in vivo [46].

Chitosan (**27**) extracted from *Aspergillus flavus* can improve the antibacterial activity of CAZ. The results showed that *P. aeruginosa* was treated with CAZ alone, the inhibition zone diameter was 21 mm, and the MIC value was 1024 μg mL^−1^. However, the MIC of CAZ after adding chitosan changed from 1024 μg mL^−1^ to 128 μg mL^−1^, and the inhibition zone diameter expanded to 28 mm. Similarly, *S. aureus* was treated with CAZ alone, the inhibition zone diameter was 18 mm, and the MIC value was 512 μg mL^−1^. After the addition of **27**, the MIC of CAZ decreased to 64 μg mL^−1^, and the diameter of the inhibition zone was also expanded to 24 mm. In addition, **27** could also be observed using scanning electron microscopy to inhibit the biofilm formation of *P. aeruginosa*, indicating that **27** has the potential to be used as an antibiotic adjuvant [48].

Copper (II) aromatic nitrogen heterocyclic complex **28** has potential QSI activity. In order to explore whether **28** has synergistic antibacterial activity, the *P. aeruginosa* clinical isolate DM-18 was treated with **28** combined with antibiotics at different concentrations. The results showed that the MIC value of CAZ decreased by 2-fold (from 1000 μg mL^−1^ to 500 μg mL^−1^) after **28** (500 μg mL^−1^) combined with CAZ, and the MIC of CAZ decreased by 4-fold (from 1000 μg mL^−1^ to 250 μg mL^−1^) after **28** (1000 μg mL^−1^) combined with CAZ. This suggests that this combination therapy is likely to become a new method of fighting bacterial infections and may have wider medical applications in the future [49].

**Figure 6:**
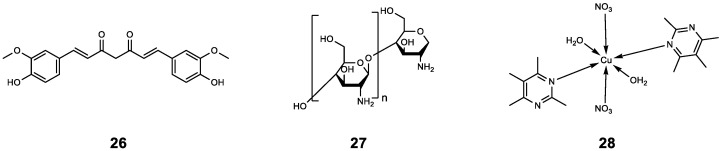
The chemical structures of compounds **26**–**28**.

The above results indicate that CAZ and QSIs have the potential for combined application. In particular, this study not only clearly illustrates the synergistic mechanism of tryptophan-containing peptides L1W and L12W combined with CAZ, but also shows that **27** and **28** can reduce the MIC of CAZ, which is likely to mean that fewer doses of CAW can exert better antibacterial effects while reducing the possibility of drug resistance. The chemical structures of compounds **26**–**28** (Figure 6) and the results of their combined antibacterial effects with CAZ are summarized in Table 3.

### 3.2. Combination of Meropenem and QSIs

*Vibrio harveyi* (*V. harveyi*) also has a QS system. Using *V. harveyi* BB170 as the reporter strain, scholars preliminarily screened 14 compounds, and STR7410 (**29**) had higher inhibitory activity (IC_50_ = 0.3724 ± 0.1091 μM). Its target is the AI-2 receptor protein LuxP. Subsequent studies have shown that Meropenem trihydrate (MEPM) combined with **29** exhibits a notable inhibiting effect on the biofilm mixed of *P. aeruginosa* PAO1 and *S. aureus* ATCC 25923 cells. Compound **29** considerably raised the susceptibility of biofilm cells to the MEPM. In the face of *C. elegans* after bacterial infection, the survival rate of **29** combined with the MEPM treatment group was 12.67% and 10.67% higher than that of **29** alone or MEPM alone, respectively. The above in vitro anti-biofilm experiments and in vivo anti-infection experiments have demonstrated the potential of **29** as an antibacterial adjuvant [50].

A series of 3-amino-2-oxazolidinone compounds were designed and synthesized using the oxazolidinone compound ZS-12 as the lead compound. Among them, YXL-13 (**30**) had the most significant inhibitory effect on the biofilm formation and virulence factors of *P. aeruginosa* PAO1 (IC _50_ = 3.686 ± 0.5790 μM). Subsequently, the combined antibacterial effect of **30** with antibiotics was further explored. According to the findings, the MIC for the combined group was 8-fold lower than the MIC for the MEPM group alone. Compound **30** not only restored the bactericidal activity of MEPM against *P. aeruginosa* PAO1 biofilm cells (FICI < 0.5) but also made biofilm cells more susceptible to MEPM. Therefore, the new oxazolidinone compound **30** combined with antibiotics will likely become a new method used to combat bacterial resistance [51].

**Figure 7:**
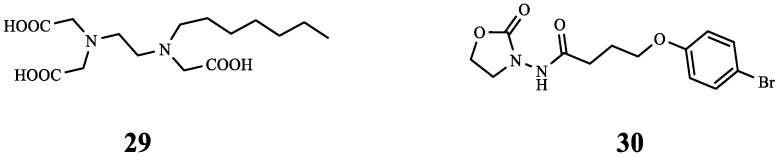
The chemical structures of compounds **29** and **30**.

The above studies not only illustrate the synergistic effect of **30** and MEPM against *P. aeruginosa* PAO1, but also show the potential of **29** combined with MEPM against mixed bacterial infections; this offers fresh concepts for future multi-bacterial illness treatment. The chemical structures of compounds **29** and **30** (Figure 7) and the results of their combined antibacterial effects with MEPM are summarized in Table 4.

### 3.3. Combination of Penicillin G with QSIs

The synergistic antibacterial effect of Cur-DA NPs and TOB and the possible synergistic antibacterial mechanism has been explained previously. Here, the synergistic anti-biofilm effect of Cur-DA NPs and penicillin G (PEN) is further explained. Similarly, the biofilm biomass of *P. aeruginosa* treated with 1 mg mL^−1^ of PEN decreased to 35.3%. However, if the biofilm of *P. aeruginosa* was pretreated with Cur-DA NPs in advance and then treated with 1 mg mL^−1^ of PEN, the biofilm biomass could be reduced to 21.9%, which further proved the possibility of Cur-DA NPs to be used as a synergistic anti-biofilm agent and the bright future of nanomaterial-carrying drugs [24].

## 4. Combination of Tetracycline Antibiotics and QSIs

The SOS reaction in bacteria is the main cause of drug-resistant mutation [52]; in order to inhibit drug-resistant mutations, some SOS inhibitors have emerged [53]. In particular, studies have found that some QSIs can block SOS expression and restore bacterial sensitivity to tetracycline (TET) [54].

**Figure 8:**
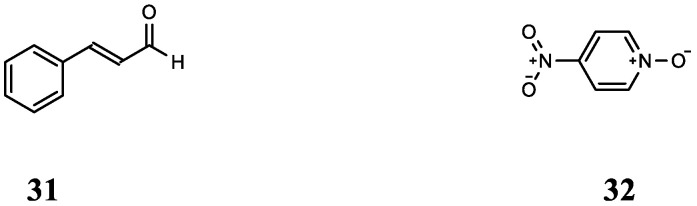
The chemical structures of compounds **31** and **32**.

**Figure 9:**
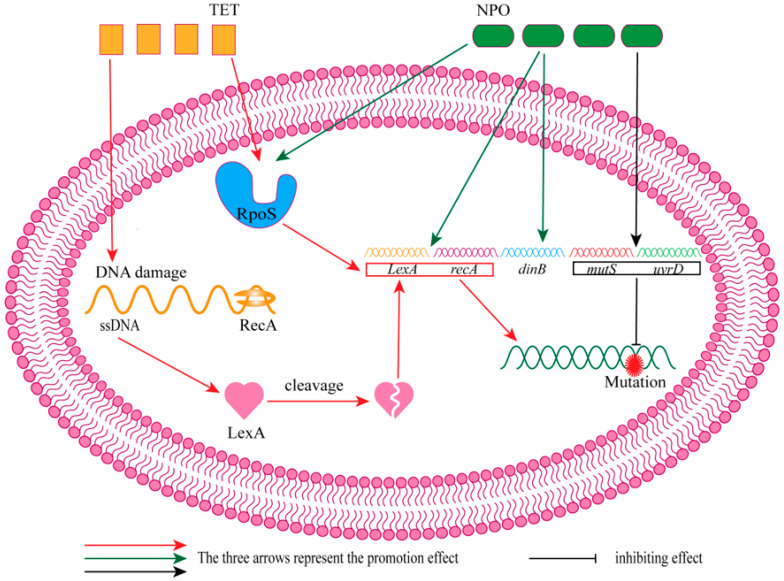
The mechanism action of NPO against TET-induced drug-resistance mutations. TET enhanced drug-resistance mutations by promoting the expression of *lexA* and *recA*, and NPO maintained drug-resistance mutations at a low level by promoting the expression of *mutS* and *uvrD*.

A better understanding of the combined effects of antibiotics and QSIs on bacteria and the specific mechanism of action is important for preventing drug resistance. Therefore, the combined effects of chloramphenicol (CHL), erythromycin (ERY), kanamycin (KAN), and TET combined with cinnamaldehyde (**31**) or 4-nitropyridine-*N*-oxide (**32**) were studied (Figure 8 shows the chemical structures of compounds **31** and **32**) in drug-resistance mutations in *Escherichia coli* (*E. coli*). First, by comparing concentration addition (CA) models and concentration–response curves (CRCs), it can be seen that, except for the additive effect of ERY + **32** and TET + **32** mixtures, other groups have weak antagonistic effects. When further exploring the effects of antibiotics and QSIs on *E. coli* resistance mutations, there was no significant difference in mutation rate between the mixtures of almost all groups and the control, indicating that QSIs may inhibit bacterial-resistance mutations. In addition, studies have found that antibiotics alone (CHL, TET), QSIs (**31**, **32**), or a combination of both will lead to mutations in the *rpoB* gene and reduce the binding force of rifampicin to the target protein RpoB. Finally, in order to investigate the mechanism of QSIs combined with antibiotics on the resistance mutation of *E. coli*, the effects of TET and **32** alone or in combination on the expression of the SOS gene (*lexA* and *recA*) and *rpoS*, *dinB*, *mutS,* and *uvrD* were studied. The findings revealed that TET could induce DNA damage and initiate the SOS response. When single-stranded DNA (ssDNA) binds to RecA, it facilitates the proteolytic self-cleavage of LexA protein, which in turn enhances the expression of *lexA* and *recA*, leading to the emergence of drug-resistant mutations. In addition, TET acting on RpoS protein can also promote bacterial resistance mutations; **32** can also up-regulate the expression of *lexA* and *recA* and act on RpoS, but it did not lead to the occurrence of *E. coli* resistance because it promoted the expression of *dinB*, *mutS*, and *uvrD* (DNA repair) simultaneously. Although the mixture of the two can promote the expression of the *lexA* gene, the **32** in it can promote DNA repair by promoting the expression of *mutS* and *uvrD*, thus reducing the frequency of mutations again (Figure 9 shows the mechanism action of NPO against TET-induced drug resistance mutations). The results showed that combining antibiotics and QSI may be a new method used to combat bacterial resistance. However, whether the attenuation effect of QSI on drug resistance mutations is universal remains to be further studied [54].

## 5. Combination of Macrolide Antibiotics and QSIs

Macrolide antibiotics are commonly used to fight inflammation caused by respiratory tract infections. However, with the emergence of drug-resistant bacteria, macrolides have also fallen to the altar. Fortunately, the emergence of antibacterial synergists may save the status of macrolides [55]. In particular, QSIs have the potential to become antibacterial synergists, and combination therapy is likely to become a new means of combating bacterial resistance [56,57].

*Cyclo*(L-Trp-L-Ser) has been identified as a QSI of *P. aeruginosa*. Research has revealed that the cyclic dipeptide C (WS) not only enhanced activity against the *P. aeruginosa rhl* system after glycosylation (Glc) or galactosylation (Gal), but also possesses strong anti-biofilm and anti-adhesion properties. After treatment with azithromycin (AZM) at a concentration of twice the MIC for 1 h, the bacterial density of *P. aeruginosa* was still 99%. After adding galactosylated *cyclo*(L-Trp-L-Ser) [c(WS)-Gal] (**33**) or glucosylated *cyclo*(L-Trp-L-Ser) [c(WS)-Glc] (**34**), the bacterial density decreased to 81% and 73%, respectively. Moreover, the bacterial survival rate of the AZM group was still 27% after 6 h, while the bacterial survival rates of AZM combined with **33** and **34** were 7% and 5%. In addition, the study found that **33** and **34** are more likely to aggregate into nanoparticles at higher concentrations and are likely to bind to AZM through non-covalent interactions, which in turn leads to AZM being more easily approached by bacteria. This shows that glycosylated modified cyclic dipeptides provide new ideas for fighting bacterial infections, but the synergistic mechanism of these glycosylated cyclic dipeptides with antibiotics needs further exploration [56].

**Figure 10:**
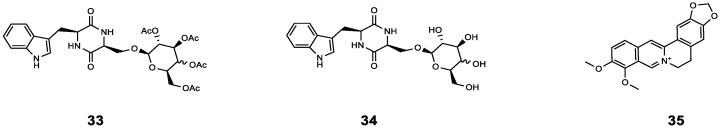
The chemical structures of compounds **33**–**35**.

Berberine (**35**), a natural isoquinoline alkaloid derived from plants, can act on the *las* and *rhl* systems of *P. aeruginosa*. Research has indicated that AZM and **35** have a synergistic effect on 10 clinical isolates and the standard reference strain *P. aeruginosa* ATCC27853; in particular, PA03 is the most susceptible due to its lowest FICI (0.13), and **35** can reduce the MIC value of AZM against PA03 (from 256 μg mL^−1^ to 16 μg mL^−1^). Moreover, the combination of AZM and **35** can inhibit the growth of *P. aeruginosa,* reduce the level of *P. aeruginosa* virulence factors, decrease the expression of *P. aeruginosa* QS-related genes, and enhance the survival percentage of *P. aeruginosa*-infected mice. In particular, the combination of the two inhibits the formation of biofilm matrix alginate and the expression of eDNA, which prevents biofilm growth and may enhance the antibacterial effect of antibiotics. The above results indicate that **35** is expected to be used as an adjuvant to enhance the antibacterial activity of AZM in vitro and in vivo. Still, its detailed synergistic mechanism remains to be studied [57]. The chemical structures of compounds **33**–**35** (Figure 10) and the results of their combined antibacterial effects with AZM are summarized in Table 5.

## 6. Combination of Quinolone Antibiotics and QSIs

As a broad-spectrum antibiotic, quinolone antibiotic CIP is often used in chronic otorhinolaryngology, endocarditis, lower respiratory diseases, etc. However, the emergence of drug resistance has led us to seek new alternatives [58]. In particular, many studies have found that CIP and QSIs have a good synergistic antibacterial effect, which may become a new way to fight bacterial infections [59,60].

The structure of QSI furanone bromide was further modified to obtain 4-(substituted phenyl)-5-methylene-2(5*H*)-furanone derivatives. Then, the combination effects of compounds **36**–**44** with subinhibitory concentration CIP were further explored. The results showed that the inhibition rate of 0.4 μg mL^−1^ CIP on the activity of *P. aeruginosa* 27853 was 26.2%. However, when combined with 256 μg mL^−1^ of **42**, the inhibition rate increased to 77.2%. Even when combined with a lower concentration of **36** (64 μg mL^−1^), the inhibition rate still reached 13%. In addition, **37**, **38**, **39**, and **41** also showed synergistic activity, which increased CIP activity by more than 1.5-fold. Similarly, the inhibition rate of CIP (0.4 μg mL^−1^) on the activity of *P. aeruginosa* PAO1 was 42.1%, while the inhibition rate was 90.53% when compound **44** or **42** was combined with 0.4 μg mL^−1^ of CIP. In particular, when 1 μg mL^−1^ of compound **42** was combined with 0.4 μg mL^−1^ of CIP, the inhibition rate still reached 60.5%. In addition, further in vivo activity preliminary exploration experiments also proved that compound **42** could successfully increase the antibacterial activity of CIP in a mouse model of PAO1 infection, which further proved the potential of QSIs combined with antibiotics against bacterial infection [61].

The synergistic antibacterial effect of **6** and **7** combined with TOB has been discussed previously. Similarly, **6** can lead to a 200–1000-fold increase in the synergistic antibacterial efficacy of CIP [22]. Compound **7** can also be used as an antibacterial synergist for CIP. During in vitro activity experiments, CIP (1/2-1/8 MIC) combined with **7** treatments inhibited the activity of *P. aeruginosa* PAO1 and three clinical isolates (FB, 1121 and 1129) more than CIP treatment alone. During in vivo studies, untreated nematodes only survived for 24 h, and **7** treatments (10 and 50 μM) alone could prolong the survival time of nematodes to 30 h and 36 h, respectively. CIP treatments alone could reduce the mortality of nematodes, resulting in partial survival of nematodes after 48 h of infection (13.1%); interestingly, the combination of **7** (10 and 50 μM) with CIP further increased the survival rate of nematodes to 35.7% and 87.1%, respectively. These results also demonstrate the potential of **6** and **7** as an antibacterial adjuvant, providing a new option for future resistance to *P. aeruginosa* drug-resistant infections [25].

2-*tert*-butyl-1,4-benzoquinone (**45**) is a QSI of *C. violaceum* ATCC12472 (MIC = 125 μg mL^−1^). Treatment of *C. violaceum* ATCC12472 with 12.5, 25, and 50 μg mL^−1^ of **45** combined with 0.2 μg mL^−1^ of CIP can significantly reduce the survival rate of biofilm and biofilm cells compared with CIP treatment alone. Significantly, 50 μg mL^−1^ of **45** combined with 0.2 μg mL^−1^ of CIP inhibited 73.27% of biofilm formation and caused a decline in the cell survival rate in the biofilm to 26.73%. Subsequently, the biofilms of different treatment groups were stained with acridine orange and observed with CLSM. The biofilm treated by the combined treatment group is looser. Therefore, it is not difficult to speculate that **45** is likely to inhibit the biofilm formation of *C. violaceum* ATCC12472 and make it easier for CIP to enter bacteria to exert antibacterial effects, which indicates the potential of **45** as an antibacterial synergist for CIP [62].

Previous studies have shown that the 3-hydroxypyridin-4(1*H*)-one nucleus has a core skeleton for anti-biofilm. Based on this skeleton, more 3-hydroxypyridin-4(1*H*)-one heterocyclic compounds can be obtained by further modification. Among them, the compound with the best biofilm inhibitory activity of *P. aeruginosa* is **46** (IC_50_ = 10.59 ± 1.17 μM). Research indicates that the MIC values of *P. aeruginosa* PAO1 and multidrug-resistant strains (1121, FB) treated with CIP alone are about 0.5 μM, 0.5 μM, and 1.0 μM, respectively. However, when combined with 20 μM **46**, the MIC value of CIP can be reduced by half. This shows that QSIs combined with antibiotics are anticipated to become a new measure against drug-resistant biofilms [63].

Studies have shown that the combination of LasR antagonists with QS signal molecules and CIP can obtain a compound ET-37 (**47**) with better QS activity, and the antibacterial effect of **47** combined with antibiotic CIP on *P. aeruginosa* (clinical strains, wild strains, and LasR mutant strains) was investigated. The results showed that only clinical strains were found to be resistant strains, and further tolerance studies found that the minimal duration for killing 99% (MDK_99_) of clinical strains treated with CIP alone was 24 h. In comparison, the MDK_99_ of wild strains and LasR mutant strains treated with **47** alone was 12 h. This further indicates that the decrease in the probability of tolerant bacteria after **47** treatments may be related to changes in gene expression. Moreover, **47** can promote the accumulation of CIP in cells and increase the concentration of superoxide anion (O_2_^•−^) in cells, resulting in succinate-semialdehyde dehydrogenase/methylmalonate semialdehyde dehydrogenase being unable to buffer oxidative stress, thus aggravating bacterial damage. The above studies indicate that combining the structure of QSIs and CIP is an important means to obtain new drugs. By affecting the formation of bacterial biofilms in patients with chronic infection, it is likely to reduce the occurrence of bacterial resistance to antibiotics [64].

Terpinen-4-ol (**48**) is a terpene compound extracted from tea tree oil. Studies have shown that after CIP being combined with **48**, the MIC values of **48** and CIP were reduced by 8-fold and 4-fold, respectively, compared with CIP alone. The two had a strong synergistic effect against *P. aeruginosa* (FICI = 0.36). In addition, time-kill analysis, biofilm formation, and eradication experiments have demonstrated the potential of **48** as an antibacterial synergist for CIP. However, the synergistic mechanism of the two is not clear, but **48** likely causes the loss of bacterial biofilm integrity, interferes with DNA replication and protein synthesis, and indirectly improves the antibacterial effect of CIP [65].

The synergistic antibacterial potential of the oxazolidinone derivative **30** with MEPM has been previously illustrated. Here, a series of benzoxazolone derivatives were further designed and synthesized, and the synergistic antibacterial effects of multiple compounds (**49**–**55**) with antibiotics were explored. The results showed that the combination of **49**–**55** (32 μg mL^−1^) with CIP (0.2 μg mL^−1^) or CLA (3.2 μg mL^−1^) significantly increased the mortality of *P. aeruginosa* PAO1. Among them, compound **52** had the best effect. When **52** was combined with CIP or CLA, the mortality of *P. aeruginosa* was 48.27% and 49.79%, respectively; however, the mortality rates of CIP or CLA alone against *P. aeruginosa* were 16.99% and 29.11%, respectively. The above experiments have shown the potential of **49**–**55** as an antibacterial synergist, but more in vitro and in vivo experiments may be needed to further illustrate the synergistic effect of the two [66].

Founded on the finding that PqsR antagonist and PqsD inhibitor work together to reduce pyocyanin, a low-molecular-weight and highly soluble dual inhibitor pyrimidine compound **56** was created. Studies have shown that **56** significantly inhibited the release of eDNA (7% ± 2 residual eDNA was detected) and caused the polysaccharides (PS) and protein BF components to be significantly attenuated to 27% ± 20 and 25% ± 13, respectively. Under biofilm conditions, CIP almost did not inhibit the activity of *P. aeruginosa*. However, with **56**, antibiotic activity could be restored under simulated biofilm conditions of chronic infection in vitro. This may be because **56** leads to decreased eDNA targeting, which leads to higher antibiotics efficacy. The above results indicate that the dual inhibitor **56** has a good application prospect, and **56** combined with antibiotics brings great hope for treating of acute and chronic *P. aeruginosa* infections in the future [67].

Hordenine (**57**) is an alkaloid found in plants. The effect of **57** and CIP on *Serratia marcescens* NJ01 biofilm was analyzed using scanning electron microscopy (SEM) and CLSM images. The results demonstrated that more than 50% of biofilm biomass and sessile cells were eliminated when exposed to **57** (25–100 μg mL^−1^) and 0.3 μg mL^−1^ CIP (MIC, 0.5 μg mL^−1^); moreover, **57** significantly increased the susceptibility of pre-formed biofilms to CIP by lowering the synthesis of EPS, damaging structural integrity of biofilms, and altering the permeability of biofilm. In particular, the biofilm thickness treated with **57** combined with CIP decreased from 13.40 ± 3.38 μm to 4.07 ± 1.49 μm. Compound **57** at concentrations of 25, 50, and 100 μg mL^−1^ resulted in EPS reductions of 35, 50, and 70%, respectively. This indicates that **57** is promising as an antibacterial adjuvant to reduce the dose of antibiotics and reduce the risk of antibiotic resistance [68].

**Figure 11:**
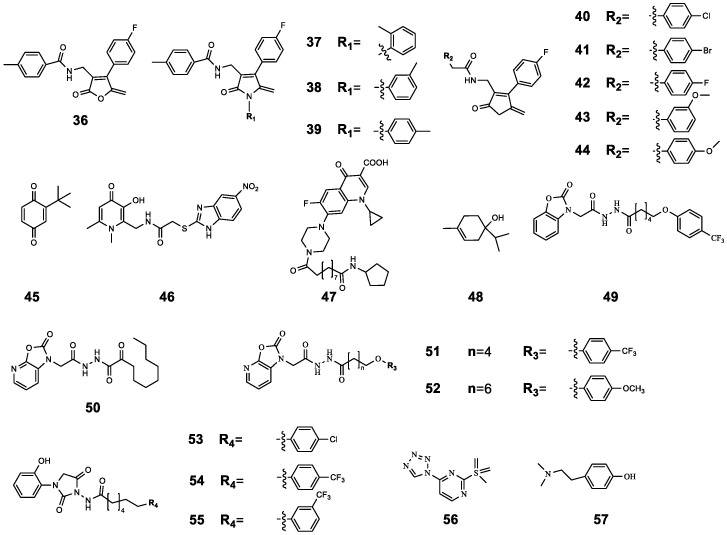
The chemical structures of compounds **36**–**57**.

At present, the research results of CIP combined with QSIs against bacterial infection are relatively optimistic, but the research is not comprehensive enough and rarely involves in vivo studies. In addition, there is no systematic study on the synergistic antibacterial mechanism of the two, which may be a topic for further discussion. The chemical structures of compounds **36**–**57** (Figure 11) and the results of their combined antibacterial effects with CIP are summarized in Table 6.

## 7. Combination of Polymyxin and QSIs

Compared with the previous several antibiotics, polymyxin is applied relatively less because it can lead to human nephrotoxicity and neurotoxicity [69]. However, if the QSIs are combined with polymyxin, it is likely to achieve a multiplier effect and reduce the generation of toxic side effects [70].

Previous studies have found that isoxazole and 1,2,3-triazole compounds have antibacterial activity, and β-thymidine derivatives with lactam structure have QSI activity. The introduction of isoxazole and 1,2,3-triazole into thymidine derivatives resulted in compounds **59**, **60**, and **61** with better activity, and then their combined effects with antibiotics were further discussed. The results showed that the combined use of colistin sulfate (polymyxin E) and compounds **59**, **60**, and **61** was significantly better than the use of antibiotics alone; when polymyxin E was used alone, the log CFU/biofilm was close to 15, but when polymyxin E was used in combination with **59**, **60**, and **61**, the log CFU/biofilm was lower. In addition, contrasted with the control group, the survival rates of *P. aeruginosa* PAO1-infected nematodes treated with **61** or TOB alone were 18.9% and 33.9%, respectively, but the combination of **61** and TOB increased the survival rate to 55.8%. The above results not only provide a new method for the discovery of antibacterial drugs, but also show the potential of **59**, **60**, and **61** to be used as antibacterial adjuvants [70,71].

Norharmane (**58**) was isolated from the fermentation broth of *Microbactrium* sp. 40DY182. When **58** was combined with polymyxin B (PB) to treat MDR *P. aeruginosa*, there was a synergistic effect (FICI = 0.266). In addition, the in vivo efficacy of the combination therapy was evaluated using a mouse model of MDR *P. aeruginosa* infection, a type that closely resembles lung infection in humans. The results showed that the **58**-PB combination caused a 1~2 order of magnitude reduction in the amount of bacteria in the affected tissue, compared with 10 mg/kg/day of PB and 20 mg/kg/day of PB. In addition, treatment with 15 mg/kg/day of **58** combined with 10 mg/kg/day of PB and 30 mg/kg/day of **58** combined with 20 mg/kg/day of PB caused the bacterial load to drop by roughly 31-fold and 245-fold, respectively, and did not increase the cytotoxicity of kidney and liver in mice. Furthermore, **58**-PB did not affect IL-12 while dramatically lowering TNF-α, IL-1β, and IL-23; this suggests that **58** may enhance the susceptibility of PB to pathogenic T_H_17 cells, thereby reducing tissue damage. These results suggest that the combination of **58** and PB may be a promising method to control the outbreak of drug-resistant bacteria. However, whether it can be used in clinical practice remains to be studied [71].

**Figure 12:**
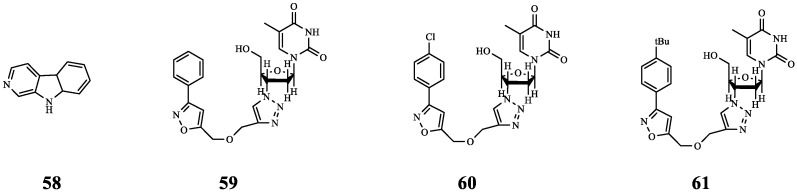
The chemical structures of compounds **58**–**61**.

There are few examples of QSIs combined with polymyxin against bacterial infections, but the above research also involves in vivo experiments, making the results more convincing. In particular, the study of **58**-PB is more in depth, not only proving the synergistic effect of the two in vivo and in vitro, but also proving the low toxicity of the combination, which provides a basis for further clinical research. The chemical structures of compounds **58**–**61** (Figure 12) and the results of their combined antibacterial effects with polymyxin are summarized in Table 7.

## 8. Combination of Other Antibiotics and QSIs

Unlike the mainstream antibiotics described above, relatively few studies have been conducted on the combination of fusidic acid (FA) or clindamycin (CD) with QSIs. However, this drug combination provides a new direction for the fight against *S. aureus* or its resistant strains [72,73,74].

In order to explore whether Furvina (**62**) and its structural analogue **63** can increase biofilm susceptibility to FA, *S. aureus* ALC1742 and *S. aureus* ALC1743 were treated with **62** or **63** combined with FA, respectively. The results showed that the combined use of FA and **62** reduced the biofilm mass of *S. aureus* ALC1742 by 38% and the biofilm mass of *S. aureus* ALC1743 by 29%; however, the use of FA alone only reduced the biofilm mass by 20%. Similarly, the combination of FA and **63** reduced the biomass of *S. aureus* ALC1742 by 34%, but did not affect *S. aureus* ALC1743. Considering the reduced metabolic activity, the inactivation rate of **63** combined with FA was 80%, notably higher than that of FA alone or combined with **62** (~70%). In addition, for strains and different concentrations of **63** and **62**, most bacteria maintain membrane integrity (more than 70%), which is essential for the effective treatment of bacterial infections. Nevertheless, the synergistic mechanism between FA and **62** or **63** remains to be understood [72].

Based on the anti-inflammatory drug Diflunisal, 16 aza analogues of *S. aureus* virulence factor inhibitors were synthesized. The most active compound, Azan-7 (**64**), showed a synergistic effect with clindamycin (CD); the FICI was 0.45, resulting in a significant decrease in the CFU of Synergize with CD against Methicillin-Resistant *S. aureus* (MRSA). In addition, the addition of **64** to CD at different time points after biofilm formation significantly enhanced the effect of CD on biofilm; the results above show that compound **64** is a promising candidate for the treatment of MRSA infection, and has a synergistic anti-MRSA effect when combined with CD. However, the current research is limited to in vitro studies, so the synergistic antibacterial effect of the two can be further investigated through in vivo experiments [73].

*Salvia tingitana* yields the labdane diterpenoids sclareol (**65**) and manool, which may be used as QSIs to combat MRSA. To explore whether **65** has a synergistic effect with CD, the researchers established a chessboard analysis to calculate the FICI. The results showed that **65** has a synergistic effect with CD (FICI was 0.45); the study also found that **65** can bind to the AgrA protein, which may disable AgrA signal receptors. Once the AgrA signal receptor is disabled, it inhibits the release of virulence factors of *S. aureus*, which also shows that **65** binding to CD can effectively inhibit the QS system in bacteria and treat bacterial infection [74].

**Figure 13:**
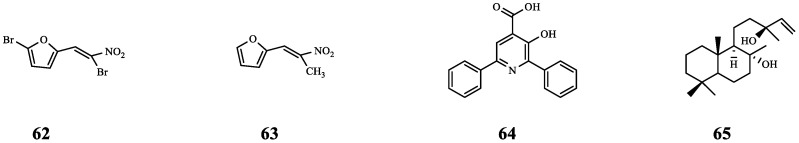
The chemical structures of compounds **62**–**65**.

In addition to the antibiotics and QSIs mentioned above, it is believed that more people will pay attention to combined treatment in the future, and researchers will progress on the road to fighting bacterial resistance. The chemical structures of compounds **62**–**65** (Figure 13) and the results of their combined antibacterial effects with antibiotics are summarized in Table 8.

## 9. Conclusions

The problem of drug resistance caused by antibiotic abuse is still an important problem that researchers need to solve [75]. Most of the research results in this review illustrate that QSIs are likely to improve the antibacterial effect of antibiotics by inhibiting and eliminating bacterial biofilm barriers. In particular, the synergistic inhibition mechanism of compound **12** combined with TOB on QS gene expression and virulence factors of *P. aeruginosa* was revealed [25]. In addition, we also found that compound **6**, Cur-DA NPs, can not only inhibit the QS system, but also inhibit the expression of drug efflux genes, improving the antibacterial activity of antibiotics [22,24]. L1 W and L12 W can not only inhibit the efflux pump gene, but also inhibit β-lactamase and the decomposition of β-lactam antibiotics to improve the latter’s utilization rate [46]. Mutations in bacteria can also cause bacterial drug resistance [76]. TET + **32** can inhibit the drug-resistance mutation, providing more opportunities for the fight against bacterial drug resistance [54]. However, the current research on combining QSIs and antibiotics is not universal and in depth. In the future, the interaction mechanism and the best applicable conditions for the two can be explored with in vivo and in vitro experiments to provide new theories and means for future clinical applications.

## Figures and Tables

**Table 1:** Summary of the combination of TOB and QSIs.

Compound	Name	Target/Targeted Bacteria	Antibiotic	Combined Results	Ref.
**1**	farnesifrol A	PqsR	TOB	Compared with TOB treatment alone, the sterilization efficiency of TOB combined with **1** was increased by 59%.	[21]
**2**	farnesifrol B	PqsR	TOB	Compared with TOB treatment alone, the sterilization efficiency of TOB combined with **2** was increased by 59%.	
**3**	farnesifrol C	PqsR	TOB	Compared with TOB treatment alone, the sterilization efficiency of TOB combined with **3** was increased by 51.4%.	
**4**	gummosin	PqsR	TOB	Compared with TOB treatment alone, the sterilization efficiency of TOB combined with **4** was increased by 19.4%.	
**5**	4-farnesyloxycoumarin	PqsR	TOB	Compared with TOB treatment alone, the sterilization efficiency of TOB combined with **5** was increased by 17%.	
**6**	*N*-Hydroxy-7-((6-methyl-2-oxo-2*H*-chromen-4-yl) oxy)- heptanamide	LasR PqsR FpvA	TOB	Compound **6** increased TOB activity by 200-fold by inhibiting biofilm formation and efflux pump gene expression.	[22]
**7**	3-Hydroxy-1,6-dimethyl-2-((((1-(4-phenoxyphenyl)-1*H*-1,2,3-triazol-4-yl) methyl) amino) methyl) pyridin-4(1*H*)-one	PqsR	TOB	Compared with TOB treatment alone, the combination of **7** (10 μM and 50 μM) with TOB inhibited the activity of *P. aeruginosa*, and increased the survival rate of infected *C. elegans* by 28.6% and 64.2%, respectively.	[25]
**8**	-	PqsR	TOB	Compared with TOB alone, the antibacterial activity increased by about 62.5%.	[26]
**9**	-	PqsR	TOB	Compared with TOB alone, the antibacterial activity increased by about 50%.	[26]
**10**	-	PqsR	TOB	Compared with TOB alone, the antibacterial activity increased by about 37.5%.	
**11**	-	PqsR	TOB	Compared with TOB treatment alone, the combined treatment resulted in a more than 3-fold reduction in the total number of CFU.	[28]
**12**	-	RhlR	TOB	Synergistic effect (FICI = 0.39).	[29]
**13**	Furanone C-30	*P. aeruginosa*	TOB	Compared with **13** or TOB treatment alone, the combination reduced the CFU of *P. aeruginosa* in each implant from above 10^5^ to around 10^5^.	[30,31,32]
**14**	Ajoene	*P. aeruginosa*	TOB	In the early treatment, the therapeutic effect of the combined administration group was ~100–150-fold that of the control group, but in the late treatment, the therapeutic effect of the combined administration group was weak, and the effect was only 1.3–4-fold that of the control group.	
**15**	*N*-(2-pyrimidinyl) butanamide	*P. aeruginosa*	TOB	Synergistic anti-biofilm effect.	[33]
**16**	QAL	*P. aeruginosa*	TOB	Synergistic anti-biofilm effect.	[34]
**17**	Oleanolic aldehyde coumarate	*P. aeruginosa*	TOB	Synergistic anti-biofilm effect.	[35]
**18**	-	*P. aeruginosa*	TOB	Synergistic anti-biofilm effect.	[36]
**19**	-	*P. aeruginosa*	TOB	No synergistic anti-biofilm activity.	
**20**	4-dimethylaminocinnamic acid	*C. violaceuma*	TOB	**20** and **21** decreased ethanolamine (biofilm component) and D-proline (osmotic pressure regulator), which promoted TOB to enter bacteria more easily and exert antibacterial activity.	[38]
**21**	4-methoxycinnamic acid	*C. violaceuma*	TOB		
**22**	Albendazole	CviR LasR	TOB	Synergistic anti-biofilm effect.	[39]

**Table 2:** Summary of the combination of GM and QSIs.

Compound	Name	Target/Targeted Bacteria	Antibiotic	Combined Results	Ref.
**23**	Plumbagin	*P. aeruginosa* MTCC 424	GM	Synergistic effect (FICI = 0.192).	[40]
		*P. aeruginosa* MTCC 2488		Synergistic effect (FICI = 0.485).	
**24**	Etrasimod	*S. aureus*	GM	Synergistic effect (FICI = 0.5).	[41]
**25**	Tannic acid	*S.* Paratyphi 3336 *S.* Typhi 950	GM	By reducing the formation of bacterial EPS (biofilm component), **25** improved the inhibitory effect of GM on *S.* Paratyphi 3336 (inhibition zone from 18.7 mm to 27.0 mm) and *S.* Typhi 950 (inhibition zone from 20.3 mm to 25.0 mm).	[42]

**Table 3:** Summary of the combination of CAZ and QSIs.

Compound	Name	Target/Targeted Bacteria	Antibiotic	Combined Results	Ref.
**26**	Curcumin	*P. aeruginosa*	CAZ	Synergistic effect (FICI = 0.26).	[47]
			CIP	Additive effect (FICI = 1.0).	
**27**	Chitosan	*P. aeruginosa*	CAZ	Compared to CAZ alone, the MIC was 8-fold smaller.	[48]
**28**	-	*P. aeruginosa*	CAZ	The MIC values of CAZ decreased by 2- fold and 4-fold after CAZ was combined with CAZ at 500 μg mL^−1^/1000 μg mL^−1^ of **28**, respectively.	[49]

**Table 4:** Summary of the combination of MEPM and QSIs.

Compound	Name	Target/Targeted Bacteria	Antibiotic	Combined Results	Ref.
**29**	STR7410	LuxP	MEPM	The combination of the two had a significant inhibitory effect on the biofilm of mixed *P. aeruginosa* PAO1 and *S. aureus* ATCC 25923 cells, and increased the survival rate of infected *C. elegans* (12.67% higher than that of **29** and 10.67% higher than that of MEPM).	[50]
**30**	YXL-13	CviR	MEPM	Synergistic effect (FICI < 0.5).	[51]

**Table 5:** Summary of the combination of AZM and QSIs.

Compound	Name	Target/Targeted Bacteria	Antibiotic	Combined Results	Ref.
**33**	*cyclo*(L-Trp-L-Ser) [c(WS)-Gal]	*P. aeruginosa*	AZM	Compared with AZM treatment alone, the sterilization rate of AZM combined with **33** increased by 20%, and the bacterial density further decreased (from 99% to 81%).	[56]
**34**	*cyclo*(L-Trp-L-Ser) [c(WS)-Glc]	*P. aeruginosa*	AZM	Compared with AZM treatment alone, the sterilization rate of AZM combined with **34** increased by 22%, and the bacterial density further decreased (from 99% to 73%).	[56]
**35**	Berberine	*P. aeruginosa*	AZM	Synergistic effect (0.13 < FICI < 0.5).	[57]

**Table 6:** Summary of the combination of CIP and QSIs.

Compound	Name	Target/Targeted Bacteria	Antibiotic	Combined Results	Ref.
**36**	4-Fluoro-*N*-((4-(4-fluorophenyl)-5-methylene-2-oxo-2,5-dihydrofuran-3-yl) methyl) benzamide	*P. aeruginosa* 27853 *P. aeruginosa* PAO1	CIP	Compared with CIP treatment alone, CIP-binding compound **36**–**44** had a better antibacterial effect, and this effect was enhanced with the increase in the concentration of **36**–**44** (from 1 μg mL^−1^ to 256 μg mL^−1^).	[61]
**37**	*N*-((4-(4-Fluorophenyl)-5-methylene-2-oxo-1-(o-tolyl)-2,5-dihydro-1*H*-pyrrol-3-yl) methyl)-4-methylbenzamide	*P. aeruginosa* 27853 *P. aeruginosa* PAO1	CIP		
**38**	*N*-((4-(4-Fluorophenyl)-5-methylene-2-oxo-1-(m-tolyl)-2,5-dihydro-1*H*-pyrrol-3-yl) methyl)-4-methylbenzamide	*P. aeruginosa* 27853 *P. aeruginosa* PAO1	CIP		
**39**	*N*-((4-(4-Fluorophenyl)-5-methylene-2-oxo-1-(p-tolyl)-2,5-dihydro-1*H*-pyrrol-3-yl) methyl)-4-methylbenzamide	*P. aeruginosa* 27853 *P. aeruginosa* PAO1	CIP		
**40**	4-Chloro-*N*-((4-(4-fluorophenyl)-5-methylene-2-oxo-2,5-dihydrofuran-3-yl) methyl) benzamide	*P. aeruginosa* 27853 *P. aeruginosa* PAO1	CIP		
**41**	4-Bromo-*N*-((4-(4-fluorophenyl)-5-methylene-2-oxo-2,5-dihydrofuran-3-yl) methyl) benzamide	*P. aeruginosa* 27853 *P. aeruginosa* PAO1	CIP		
**42**	4-Fluoro-*N*-((4-(4-fluorophenyl)-5-methylene-2-oxo-2,5-dihydrofuran-3-yl) methyl) benzamide	LasR RhlR PqsR	CIP		
**43**	*N*-((4-(4-Fluorophenyl)-5-methylene-2-oxo-2,5-dihydro-furan-3-yl) methyl)-3-methoxybenzamide	*P. aeruginosa* 27853 *P. aeruginosa* PAO1	CIP		
**44**	*N*-((4-(4-Fluorophenyl)-5-methylene-2-oxo-2,5-dihydro-furan-3-yl) methyl)-4-methoxybenzamide	*P. aeruginosa* 27853 *P. aeruginosa* PAO1	CIP		
**45**	2-*tert*-butyl-1,4-benzoquinone	*C. violaceum* ATCC12472	CIP	Compared with CIP treatment alone, the combined treatment significantly inhibited biofilm formation; in particular, **45** (50 μg mL^−1^) combined with CIP (0.2 μg mL^−1^) inhibited biofilm formation by 73.27% and reduced the survival rate of biofilm cells to 26.73%.	[62]
**46**	1,6-Dimethyl-2-((5-nitro-2-benzimidazolyl)-thioacetaminomethyl)-3-hydroxy-4-pyridone	LasR PqsR	CIP	The MIC value of CIP was reduced by 50% after combination.	[63]
**47**	ET-37	*P. aeruginosa*	CIP	**47** assists CIP antibacterial by destroying biofilm and promoting the oxidative stress response.	[64]
**48**	Terpinen-4-ol	LasR RhlR PqsR	CIP	Synergistic effect (FICI = 0.36)	[65]
**49**	*N’*-(2-(2-Oxobenzo[d]oxazol-3(2*H*)-yl) acetyl)-6-(3-(trifluoromethyl)phenoxy) hexanehydrazide	*P. aeruginosa* PAO1	CIP	Compared with CIP treatment alone (16.99%), the combined effect resulted in higher bacterial mortality (40% to 50%).	[66]
**50**	*N’*-(2-(2-Oxooxazolo[5,4-b] pyridin-1(2*H*)-yl) acetyl) nonanehydrazide	*P. aeruginosa* PAO1	CIP		
**51**	*N’*-(2-(2-Oxooxazolo[5,4-b] pyridin-1(2*H*)-yl) acetyl)-6(4(trifluoromethyl)phenoxy) hexanehydrazide	*P. aeruginosa* PAO1	CIP		
**52**	8-(4-Methoxyphenoxy)-*N’*-(2-(2-oxooxazolo[5,4-b] pyridin-1(2*H*)-yl) acetyl) octanehydrazide	LasR	CIP	Compared with CIP treatment alone, the combination resulted in higher bacterial mortality (from 16.99% to 48.27%).	[66]
**53**	6-(4-Chlorophenoxy)-*N*-(3-(2-hydroxyphenyl)-2,5-dioxoimidazolidin-1-yl) hexanamide	*P. aeruginosa* PAO1	CIP	Compared with CIP treatment alone (16.99%), the combined effect resulted in higher bacterial mortality (40% to 50%).	
**54**	*N*-(3-(2-Hydroxyphenyl)-2,5-dioxoimidazolidin-1-yl)-6-(4-(trifluoromethyl)phenoxy) hexanamide	*P. aeruginosa* PAO1	CIP	Compared with CIP treatment alone (16.99%), the combined effect resulted in higher bacterial mortality (about 30%).	
**55**	*N*-(3-(2-Hydroxyphenyl)-2,5-dioxoimidazolidin-1-yl)-6-(3-(trifluoromethyl)phenoxy) hexanamide	*P. aeruginosa* PAO1	CIP	Compared with CIP treatment alone (16.99%), the combined effect resulted in higher bacterial mortality (nearly 40%).	
**56**	-	PqsR PqsD	CIP	**56** increased the antibacterial activity of CIP by inhibiting the release of eDNA and reducing the content of polysaccharide (PS) and protein BF.	[67]
**57**	Hordenine	*Serratia marcescens* NJ01	CIP	**57** significantly increased the susceptibility of pre-formed biofilms to CIP by reducing the synthesis of EPS and destroying the structural integrity of biofilms.	[68]

**Table 7:** Summary of the combination of polymyxin and QSIs.

Compound	Name	Target/Targeted Bacteria	Antibiotic	Combined Results	Ref.
**58**	-	*P. aeruginosa*	polymyxin E	Synergistic anti-biofilm effect.	[70]
**59**	-	*P. aeruginosa*	polymyxin E	Synergistic anti-biofilm effect.	
**60**	-	*P. aeruginosa*	polymyxin E	Synergistic anti-biofilm effect.	
**61**	Norharmane	PqsA	PB	Synergistic effect (FICI = 0.266).	[71]

**Table 8:** Summary of the combination of other antibiotics and QSIs.

Compound	Name	Target/Targeted Bacteria	Antibiotic	Combined Results	Ref.
**62**	Furvina	*S. aureus* ALC1742	FA	Compared with FA alone, the ALC1742 biofilm was further reduced (from 20% to 38%) after combination.	[72]
		*S. aureus* ALC1743		Compared with FA alone, the ALC1743 biofilm was further reduced (from 23% to 29%) after combination.	
**63**	-	*S. aureus* ALC1742	FA	Compared with FA alone, the ALC1742 biofilm was further reduced (from 20% to 34%) after combination.	
		*S. aureus* ALC1743		There is no effect on biofilm.	
**64**	Azan-7	MRSA	CD	Synergistic effect (FICI = 0.45).	[73]
**65**	Sclareol	MRSA	CD	Synergistic effect (FICI = 0.45).	[74]

## Data Availability

Not applicable.
